# **Sublethal effects of nanoformulated* Mentha pulegium* L. essential oil on the biological and population growth parameters of** the **greenhouse whitefly,* Trialeurodes vaporariorum,* (Hemiptera: Aleyrodidae)**

**DOI:** 10.1038/s41598-024-78249-x

**Published:** 2024-11-09

**Authors:** Mohammad Sharifiyan, Fariba Mehrkhou, Maryam Negahban

**Affiliations:** 1 Department of Plant Protection, Faculty of Agriculture, Urmia University, Urmia, Iran; 2 Research Department of Pesticides, Iranian Research Institute of Plant Protection (IRIPP), Tajrish, Iran

**Keywords:** *Mentha pulegium*, Nanoformulaion, Greenhouse whitefly, Bioassays, Two-sex life table, Ecology, Engineering, Nanoscience and technology

## Abstract

We evaluated the toxicity and sublethal effects of essential oil (*Mentha pulegium* L.) and its nanoformulation against greenhouse whitefly, *Trialeurodes vaporariorum,* which is one of the most destructive pests of a wide range of crops. The essential oil was extracted from the plant by steam distillation using a Clevenger apparatus, and 14 chemical components of *M. pulegium* were identified using gas chromatography-mass spectrometry. The results illustrated that monoterpenoids were main characterized components including pulegone (%66), menthofren (%10.54), 1, 8 Cineole (%8.36), betapenin (%3.49) and limonene (%2.01). The nanoformulation was characterized using dynamic light scattering (DLS), transmission electron microscopy (TEM), and scanning electron microscopy (SEM), revealing that the particles were spherical in shape with an average size of 156.40 nm. The leaf dipping was used for the bioassays. The obtained LC_50_ and LC_25_ values of treatments indicated that the nanoformulation of essential oil (LC_50_: 2418.96 and LC_25_: 1724. 25 ppm) was more toxic than the pure of *M. pulegium* oil (LC_50_: 3223.083 and LC_25_: 779.439 ppm ppm) against greenhouse whitefly adults after 24 h. The life table data were analyzed based on the age-stage, two-sex life table theory using computer program of TWOSEX–MSChart. Also, the sublethal concentration (LC_25_) of its nanoformulation led to delaying in preadult stage and decreased the adult longevity, and fecundity compared to treatments. Moreover, the sublethal concentration of either *M. pulegium* oil or its nanoformulation affected the population growth parameters of *T.vaporariorum* compared to the control. However, the net reproductive rate (*R*_0_), intrinsic rate of increase (*r*), finite rate of increase (λ), of adults who exposed to the nanoformulation was lower than the pure form of *M. pulegium*. The overall results demonstrated that the nanoformulation of *M. pulegium* has the most lethal and sublethal effects on greenhouse whitefly compared with the pure form of essential oil which can be consider in integrated pest management program (IPM) of this pest.

## Introduction

Insect pests reduce food security by affecting crop yield and the quality of agricultural products^1^, and transmission of plant pathogens as well^2,3^.

**Fig. 1:**
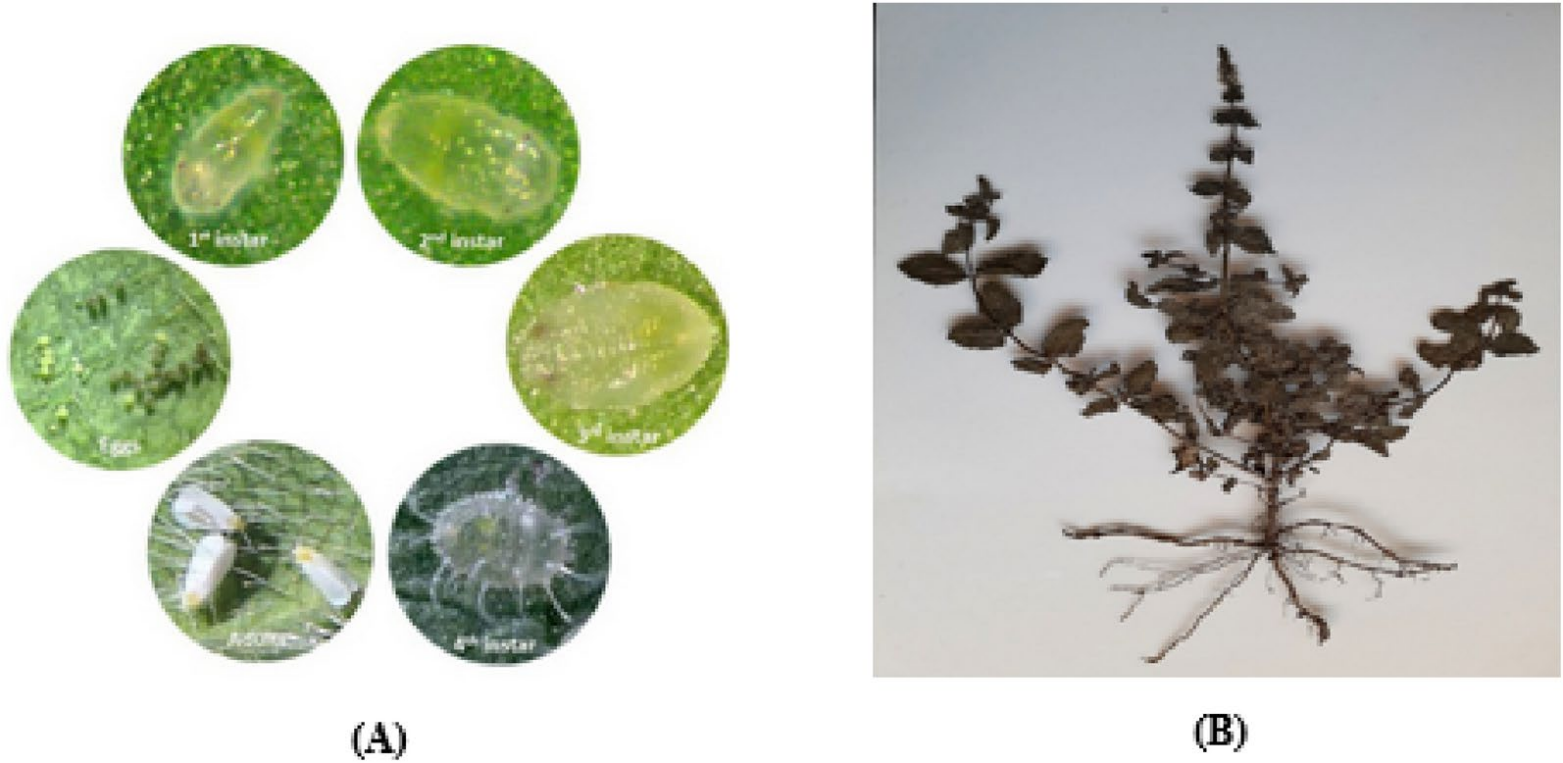
The life cycle of greenhouse whitefly, *T.vaporariorum*, (**A**) and *Mentha pulegium* (**B**).

The greenhouse whitefly, *Trialeurodes vaporariorum*, Westwood (Hemiptera: Aleyrodidae) is an economically important agricultural pest in worldwide^4,5^ (Fig. 1A). It is known as a polyphagous pest, which feeds over 275-300 different range of host plants, including vegetables, fruits and ornamental crops^6^. Either nymphs or adults cause direct damage by feeding on the plant juices and indirect damage by production of honeydew, which led to growth of black sooty mold fungus, and transmission of plant viruses as well^7–9^. The management of greenhouse whitefly is difficult owing to the polyphagous and polyvoltine behavior of greenhouse whitefly, which ultimately enable it to overcome the plant allelochemicals and resistance to synthetic insecticides^6^. Exclusive use of synthetic insecticides has led crucial side effects such as pest resistance, toxic residue in food substance, and environmental pollution^10,11^.

In recent years, entomologists have tried to find new potential substances, Essential oils (EOs), with insecticidal properties^12^. They are known as a volatile plant secondary metabolites which have various insecticidal activity against insect pests encompassing repellency, toxicity, anti-oviposition, and antifeedant activity^13–16^. Some advantages of essential oils such as eco-friendly, economic, efficiency against a variety of pests, numerous modes of action, low toxicity of residues after application, and relatively inexpensive production procedure make them a potential and suitable option^17,18^. Hence, essential oils owing to the complex mixtures of constituents and their various sites of activity on target insects, it is likely that resistance will develop more slowly against *T. vaporariorum*^19,20^. These features imply that insecticides based on plant essential oils could be used in different ways to control insect pests^21^.

*Mentha pulegium* L. as a medicinal and aromatic plant (Lamiales: Lamiaceae), has a cosmopolitan distribution^2,22^, which grows commonly in Iran^23^ (Fig. 1B). It is well known for biological properties including antimicrobial, antioxiditant, anticholinesterase and insecticidal activities^17^. The insecticidal potential and repellency activity against different insects makes it as a good source of active substances for the development of botanical insecticides^17^. However, high volatility properties, low solubility in aqueous phase and sensitivity to physical (temperature, light, ultra violet, etc) and chemical agents (oxidative and polymerization processes) of EOs resulted in a loss of their efficiency and stability^10,24,25^. Nowadays, with the development of nanotechnology, the advancement of essential oil nanoformulations have become as a promising alternative^25,26^. Due to the nanometric size of nonoformulations, the physicochemical stability and contact surface area would be increase, which led to better dispersion of EOs in aqueous phase, and increasing their interaction with the target sites^8,24,27^.

In the most studied documents, the insecticidal, antifeedant, contact and fumigant toxicity of *M. pulegium* using the lethal concentration against different insect pests such as *Drosophila melanogaster* Meigen^29^, *Aphis spiraecola* Patch, *Aphis* gossypii Fitch^30^, *Tribolium castaneum* Herbst^31^, and *Culex pipiens* Linnaeus^12^, were evaluated. There are a few information regarding the sublethal concentration of another essential oils on insect pests^9,32^. To our knowledge there is no comprehensive information regarding the sublethal effects of *M. pulegium* and its nano-formulated against whitefly greenhouse. The sublethal concentration are involved in biological, physiological, demographic or behavioral changes of individual, or populations of insects^33^. Owing to the physiological changes, prolonging in the developmental time, reduction in adult longevity, fecundity, and fertility of insect pests may occur during generations^9,12,34^. Also, the sublethal concentrations of botanical insecticides are involved in physiological processes by inhibition the ecdysis process and the acetylcholinesterase activity, as well^33^. So, besides the lethal effects, survey on the sublethal effects of *M. pulegium* and its nano-formulated can give a comprehensive insight into their use as a biopesticide in integrated pest management programs^9,35^, that could be obtain by demographic toxicological studies^34,36^, in which life tables and the projected population growth of the targeted pests could be estimate^37–40^.

To gain insight into the lethal and sublethal effects of *M. pulegium* essential oil and its nanoformulation, we used the two-sex life table method by analyzing life table and population growth rate parameters of *T. vaporariorum*. Therefore, the present study provides a comprehensive information focusing on biological (developmental time traits, adult longevity and fecundity), and population parameters such as intrinsic rate of increase, net reproductive rate, life expectancy, reproductive value and etc. of *T. vaporariorum*.

## Results

### Chemical constituents of *M. pulegium* essential oil

The chemical constituents, retention index, retention time and percentage of *M. pulegium* EO are shown in Table 1. The results indicated about 14 compounds in which, pulegone (66.00%), menthofuran (10.54%), 1, 8-cineole (8.36%), β-pinene (3.49%), limonene (2.01%), and α-pinene (1.76%) were the six most abundant constituents of *M. pulegium* EO. Their retention time were obtained 23.01, 20.17, 14.60, 12.59, 14.39 and 10.96 min, respectively.

**Table 1:** Characterization of chemical constituents of *Mentha pulegium * essential oil using GC/MS.

Components	Retention index	Retention time (min)	Percentage (%)
α-pinene	946.516	10.96	1.76
Sabinene	981.705	12.30	1.08
β-pinene	988.814	12.59	3.49
Limonene	1041.766	14.39	2.01
1,8-cineole	1047.112	14.60	8.36
*p-*menth-3-en-8-ol	1151.047	19.35	1.60
Menthone	1158.223	19.73	0.76
Menthofuran	1166.362	20.17	10.54
*cis-*dihydro carvone	1174.682	20.63	0.75
a-terpineol	1176.467	20.73	0.79
Pulegone	1257.727	23.51	66.00
Piperitenone	1350.493	27.93	0.69
Piperitenone oxide	1368.504	28.88	1.06
*E-*caryophyllene	1447.904	31.53	0.56

### Nanoformulation surface morphology, particle size distribution and encapsulation efficiency

**Fig. 2:**
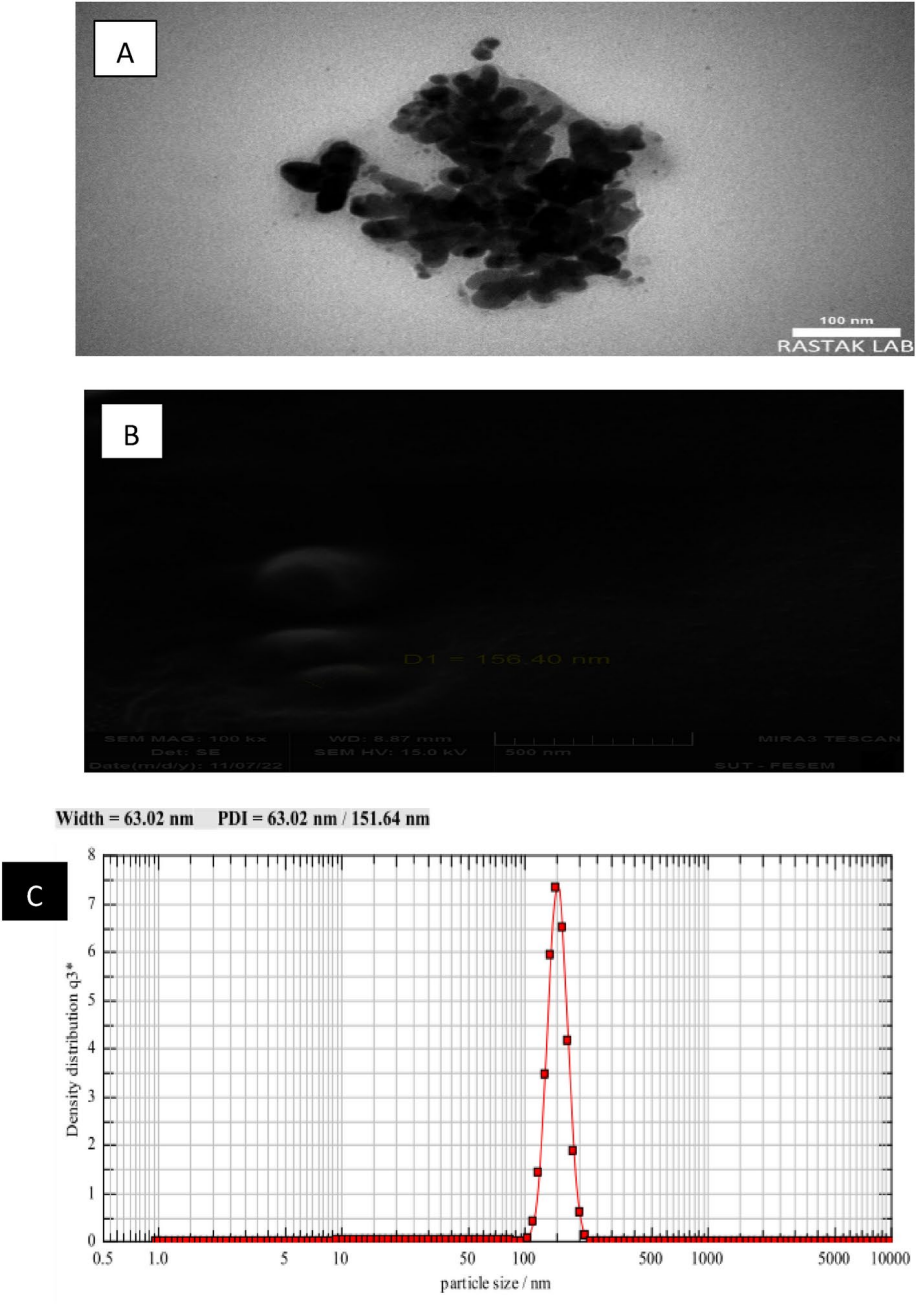
Transmission electron micrographs (TEM) (**A**), scanning electron microscopy (SEM) image (**B**), and size distribution by dynamic light scattering (DLS) of *Mentha pulegium* nanoformulation (**C**).

TEM images (Fig. 2A) show nucleus and wall structure of nanoformulation. Nanoparticle sizes by using TEM were approximately 100 nm with spherical shape. In the scanning electron microscopy (SEM) the mean size of particles were 156.40nm (Fig. 2B). Dynamic light scattering (DLS) is a technique used to measure colloidal stability through the measurement of the particle/droplet size and size distribution. The average size of particle droplet was 63.02nm (Fig. 2C). The encapsulation efficiency (EE) was obtained 88 % for *M. pulegium* of EONF.

### Insecticidal activity of *M. pulegium* essential oil and its nanoformulation

Probit analysis revealed that LC_50_ values of the pure and nanoformulation essential oil of *M. pulegium* against adults of *T. vaporariorum* 24h after treatment were 3223.083 and 2418.96 ppm, respectively (Table 2).

**Table 2:** Susceptibility of *Trialeurodes vaporariorum* adults to *Mentha pulegium* essential oil and it’s nanoeformulation 24 h after treatment.

	LC_25_ (ppm)	LC _50_ (ppm)	LC _90_ (ppm)	Slope ± SE	X^2^ (df)	P value	RMP	95% C. L.
*Mentha pulegium*	1724.526 (1208.8–2181.44)	3223.08 (2596.7–4033.75)	10576 (7512–19167)	2.483± 0.387	1.372 (3)	0.457	1.33	(1.35–1.36)
*Nanoformulation of M.pulegium*	779.439 (428.71–1098.560)	2418.968 (1922.976-2968.633)	7507.2 (5577.9–12405)	2.606±0.404	1.768	0.589		

*LC* lethal concentration, *CL* confidence limits, *X*^*2*^ Chi-square value, *df* degrees of freedom, *RMP* relative median potency.

### Sublethal effects of *M. pulegium* EO and its nanoformulation on life span of *T. vaporariorum*

The developmental time, survival, reproduction, adult longevity, and total life span of greenhouse whitefly exposed to the *M. pulegium* EO and its nanoformulation are shown in Table 3. The longest egg incubation period in the greenhouse whitefly’s F1 generation observed in the nanoformulation treatment followed by the *M. pulegium* EO, and control treatments. Exposure to sublethal concentration of *M. pulegium* EO and its nanoformulation led to prolonged developmental time of the F1 generation of the greenhouse whitefly. The developmental time duration (egg to adult emergence) obtained for the cohort in the nanoemoulsion group was significantly longer than those exposed to *M. pulegium* EO. The highest and lowest immature/preadult survival rates were obtained in the control (0.933±0.23) and *M. pulegium* nanoformulation (0.716±0.18), respectively. The longest female (8.04±0.2) and male (7.54±0.16) longevity were recorded in the control group. The effects of the *M. pulegium* EO and its nanoformulation on the total prereproductive period (TPRP), adult prereproductive period (APRP), oviposition days, as well as fecundity are shown in Table 3. Retardation in egg laying was observed in the population of adult female progeny of parents exposed to the sublethal concentration of (LC_25_). The longest and shortest APRP were obtained control (0.93±0.05 days) and nanoformulation of essential oil (0.89±0.08 days) populations, respectively (Table3).

**Table 3:** Sublethal concentrations of *Mentha pulegium* and its nanoformulation on developmental time, adult longevity and fecundity of *Trialeurodes vaporariorum*.

Stages	Control (n*)	*Mentha pulegium* (LC_25_) (n*)	Nanoformulation (LC_25_) (*)	df (F value)
Egg (days)	7.22±0.124^c^ (60)	8.04±0.15^b^ (57)	8.51±0.162^a^ (51)	238 (970.72)
Nymph (days)	11.55±0.16^c^ (56)	12.61±0.16^b^ (49)	13.02±0.14^a^ (43)	752 (3971.101)
Pupa (days)	4.12±0.09^c^ (56)	5.06±0.12^b^ (49)	5.67±0.15^a^ (43)	1298 (11516.896)
Preadult (day)	22.88±0.22^c^ (56)	25.80±0.25^b^ (49)	27.21±0.27^a^ (43)	970 (17001.099)
Preadult survival rate (%)	0.933± 0.23^a^ (56)	0.817±0.15^b^ (49)	0.716±0.18^b^ (43)	1147 (881.185)
Female longevity (days)	8.04±0.20^a^ (28)	6.33±0.16^b^ (21)	5.67±0.16^c^ (18)	372 (456.720)
Male longevity (days)	7.54±0.16^a^ (28)	5.93±0.16^b^ (28)	6.00±0.17^b^ (25)	520 (6060.004)
APRP (day)	0.93±0.05^a^ (28)	0.90±0.07^ab^ (21)	0.89±0.08^b^ (18)	67(1.384)
TPRP (day)	24.21±0.32^c^ (28)	26.52±0.32^b^ (21)	28.11±0.35^a^ (18)	1365 (8214.447)
Reproductive days (day)	5.14±0.23^a^ (28)	3.76±0.22^b^ (21)	3.06±0.19^c^ (18)	819 (4152.637)
Fecundity (eggs/♀)	25.68±1.41^a^ (28)	17.67±1.35^b^ (21)	13.44±1.40^c^ (18)	305 (1354.280)

The means followed by different letters in each row are significantly different (paired-bootstrap at 5 % significance level).

*APRP* adult prereproductive period, *TPRP* total prereproductive period.

^*^n = sample size of each stage of *Trialeurodes vaporariorum*.

**Fig. 3:**
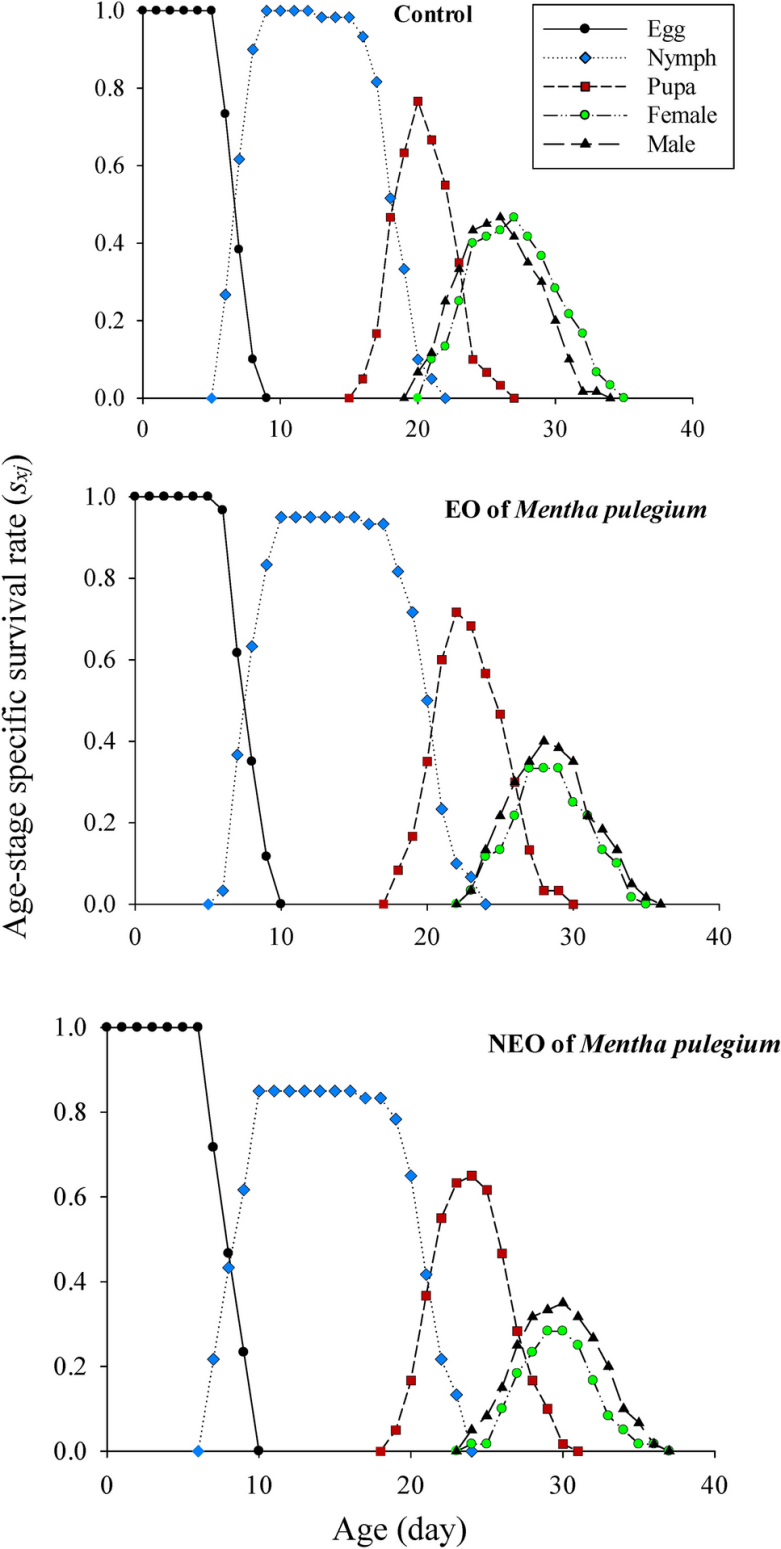
Sublethal effects of *M. pulegium* and NF of *M. pulegium* on age-stage specific survival rate (*s*_*xj*_) against *T.vaporariorum*.

**Fig. 4:**
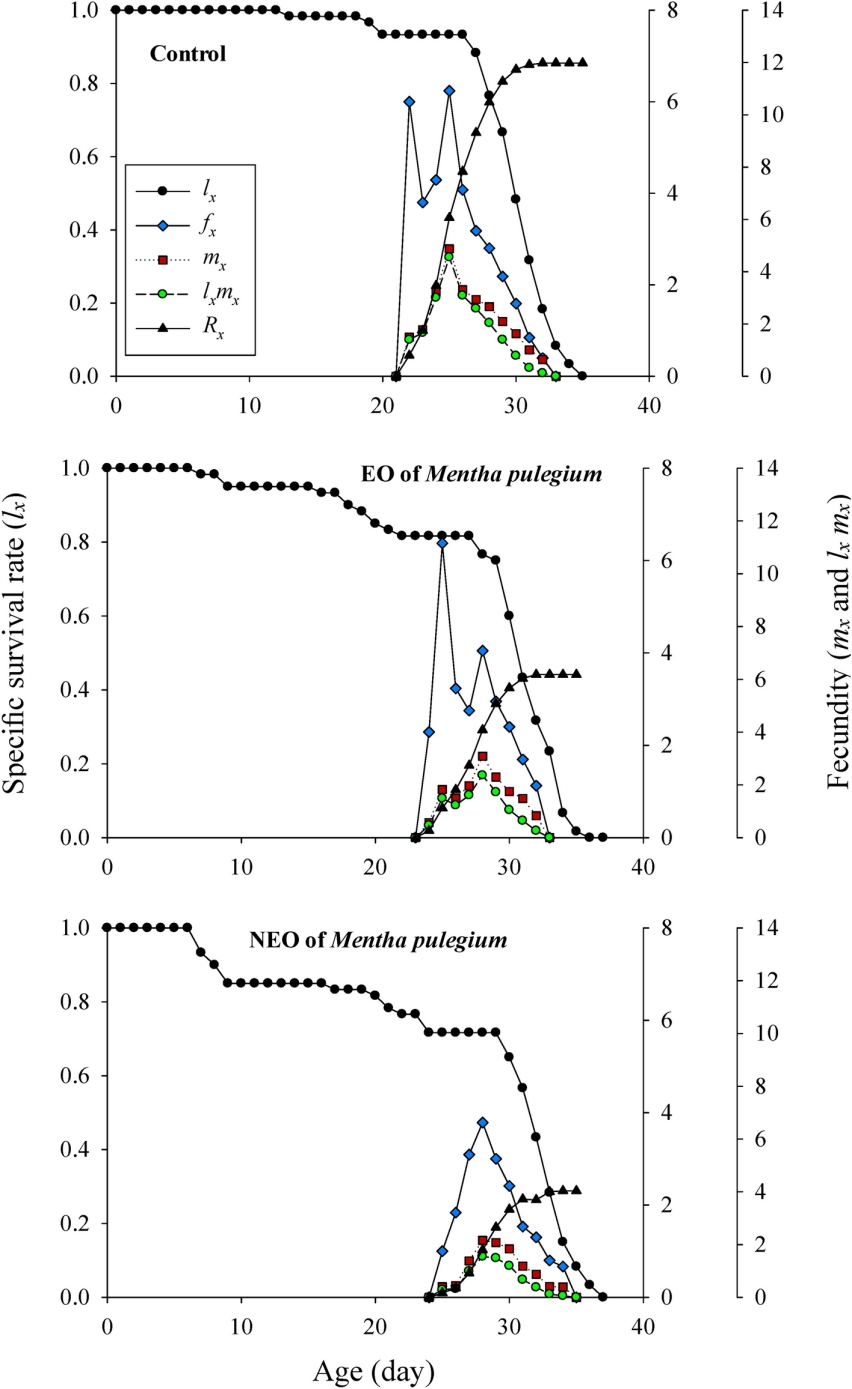
Age-specific survival rate (*l*_*x*_), female age-specific fecundity (*f*_*x*_), age-stage specific fecundity of the total population (*m*_*x*_), and net maternity (*l*_*x*_* m*_*x*_) of *T. vaporariorum* after exposure to sublethal concentration of *M. pulegium* and NF of *M. pulegium*.

**Fig. 5:**
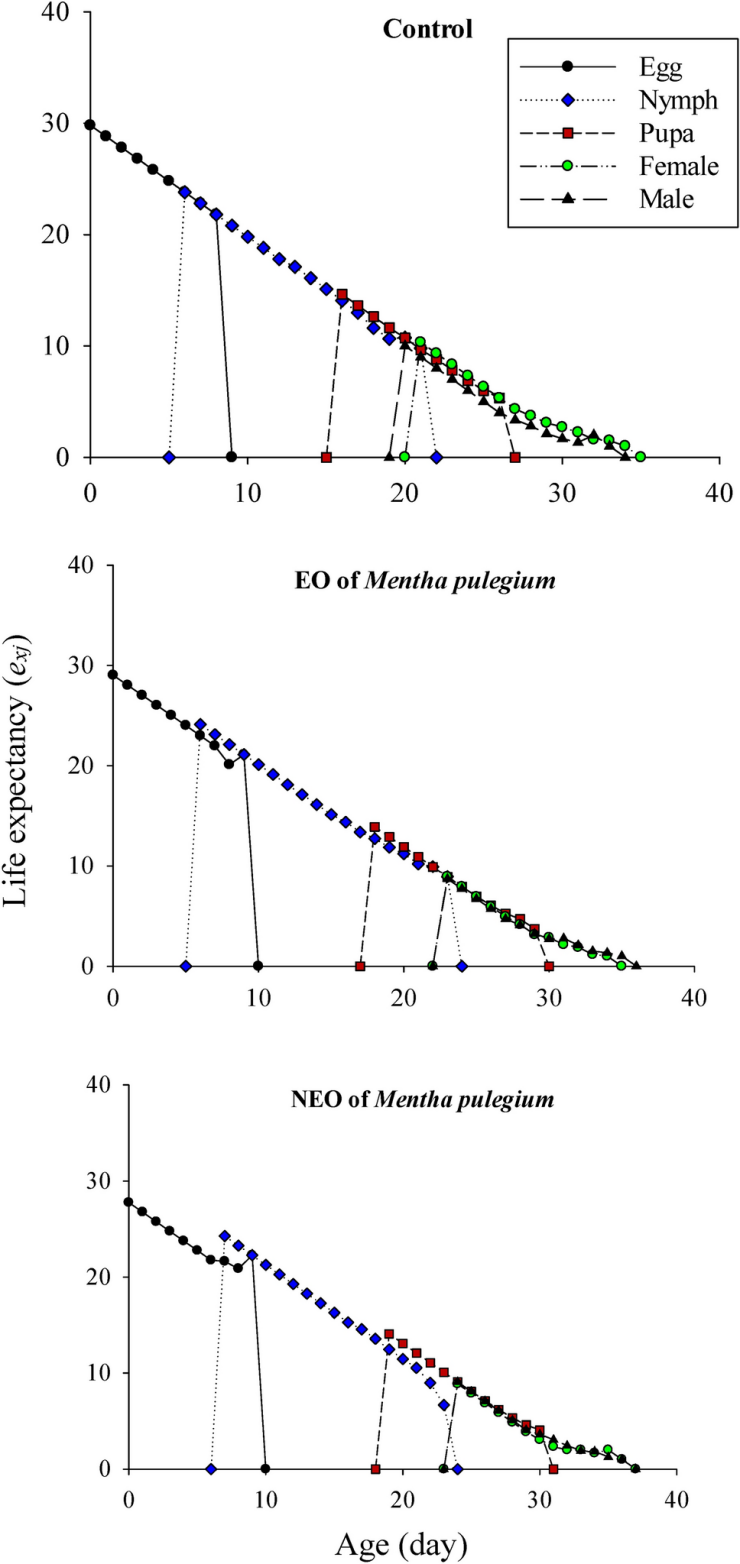
Age-stage specific life expectancy (*e*_*xj*_) of *T. vaporariorum* after exposure to sublethal concentrations of *M. pulegium* and its NF.

**Fig. 6:**
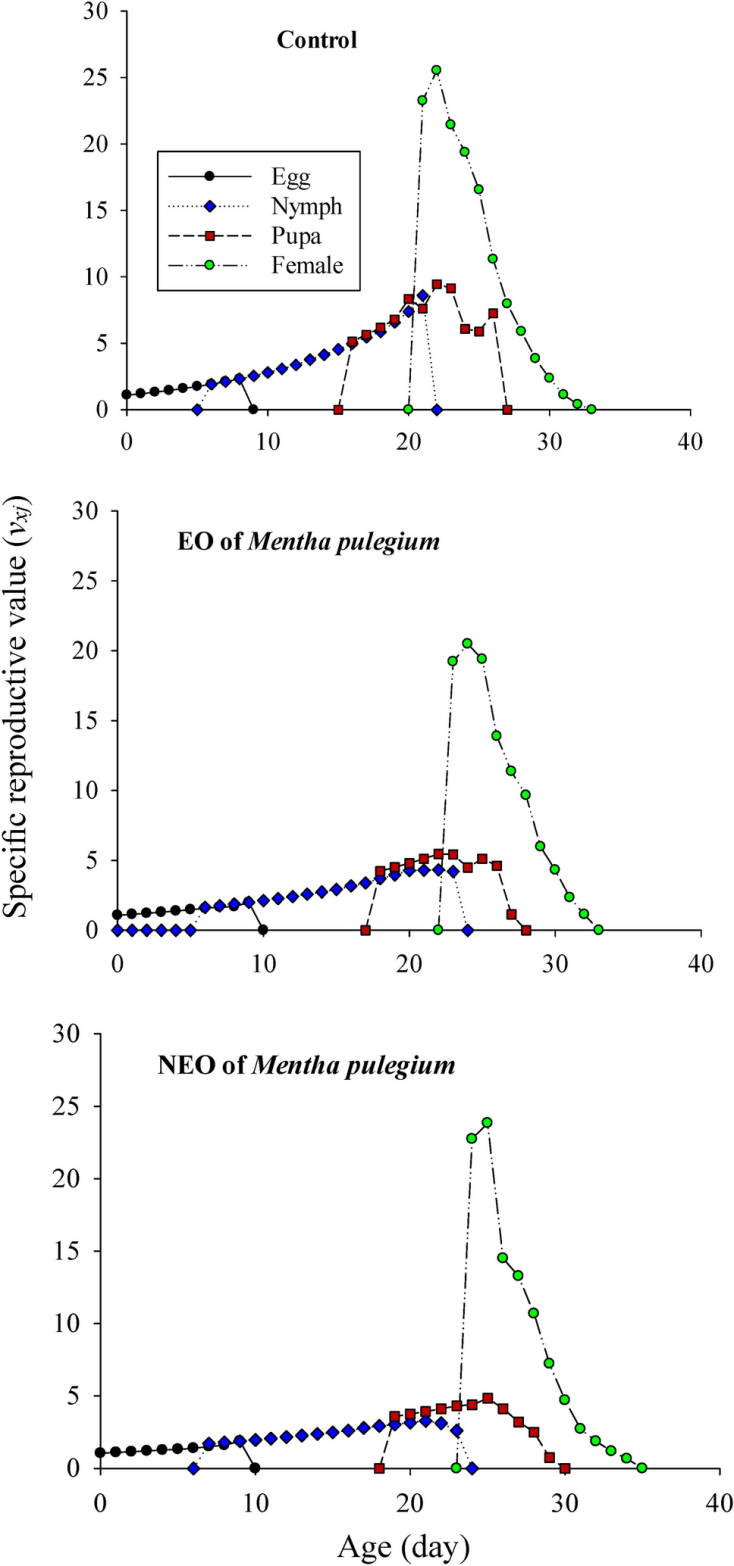
Age-stage specific reproductive value (*v*_*xj*_) of *T. vaporariorum* after exposure to sublethal concentrations of *M. pulegium* and its NF.

The sublethal effects of *M. pulegium* and its nanoemoulsion on the age-stage specific survival rate (*s*_*xj*_) of greenhouse whiteflies’ F1 generation are demonstrated in Figure 3. The survival probability of a newly born greenhouse whiteflies to adulthood was considerably lower in the nanoformulation group and the adult females emerged with delay. The age-specific survival rate (*lx*), fecundity (*m*_*x*_), maternity (*l*_*x*_*m*_*x*_), the age-stage specific fecundity (*f*_*xj*_), and cumulative net reproductive rate (*R*_*x*_) values of the greenhouse whiteflies are displayed in Figure 4. In the nanoformulation group, the appearance of the peak of the age-specific fecundity (*m*_*x*_) and age-specific maternity (*l*_*x*_*m*_*x*_) was occurred with delay and the gap between them was much higher due to the lower survival rate. Exposure to *M. pulegium* EO and its nanoformulation affected the age-stage-specific life expectancy (*e*_*xj*_) of *T. vaporariorum* (Fig. 5). The life expectancy of first day egg were 29.82, 29.03, and 27.77 days in the control, pure EO and the nanoformulation group, respectively. These results showed that the life expectancy of the greenhouse whiteflies increased with exposure to control group and decreased with exposure to treatments. The sublethal effects of *M. pulegium* and its nanoformulation on the age-stage specific reproductive value (*v*_*xj*_) of greenhouse whiteflies’ F1 generation is presented in Figure 6. The maximum *v*_*xj*_ peak values obtained in greenhouse whiteflies control cohorts were higher than that obtained in the treated group and the peak of *v*_*xj*_ occurred much later in the nanoformulation group, then followed by the pure EO, and control, respectively.

### Sublethal effects of* M. pulegium EO* and its nanoformulation on the population parameters of *T. vaporariorum*

Our results indicated that the treatment of *M. pulegium* Eo and its nanoemoulsion affected population parameters of the greenhouse whitefly. The net reproductive rate (F = 11.062; df = 3, 33; p ≤ 0.001), the intrinsic rate of increase (F = 11.720; df =3, 33; p ≤ 0.001), the finite rate of increase (F = 12.767; df = 3, 33; p ≤ 0.001), and the mean generation time (F = 3.762; df = 3,33; p ≤ 0.01) are shown in Table 4. The net reproductive rate (*R*_0_), the intrinsic rate of increase (*r*), and finite rate of increase (*λ*) were the lowest and the mean generation time (*T*) was the longest in the nanoformulation group (Table 4).

**Table 4:** The sublethal effects of *Mentha pulegium* and its nanoformulation on population growth parameters of *Trialeurodes vaporariorum*.

Population Parameters	Control (n*)	*Mentha pulegium* (LC_25_) (n*)	nanoformulation (LC_25_) ) (n*)	df (F value)
R_0_ (off spring/individual)	11.983±1.78^a^	6.183±1.18^b^	4.03±0.89^c^	1896 (3627.154)
r (d ^−1^)	0.0940±0.0060^a^	0.0640±0.0070^b^	0.0470±0.0080^c^	2073 (4889.963)
λ (d ^−1^)	1.098±0.006^a^	1.066±0.004^ab^	1.048±0.008^b^	1719 (4123.109)
T(d)	26.55±0.31^b^	28.55±0.34^ab^	29.75±0.46^a^	2250 (7305.858)
GRR (offspring/individual)	14.48±2.00^a^	8.74±1.63^b^	6.38±1.45^c^	1542 (1966.205)

The means followed by different letters in each column are significantly different (paired-bootstrap at 5 % significance level).

*R*_0_ net reproductive rate, *r* intrinsic rate of increase, *λ* finite rate of increase, *T* mean generation time, *GRR* Gross reproductive rate.

^*^n = sample size of each stage of *T. vaporariorum.*

## Discussion

Due to the side effects of synthetic insecticides, survey on the insecticidal activity of essential oils (EOs) have been increasingly in recent years. Since, the major compounds consider in the synthetize and formulation of biopesticides, essential oils^41^, so we discussed on the bioactivity of pulegone, as a major compound of *M. pulegium* on the biological traits of greenhouse whitefly. In the current study, pulegone was obtained with the most abundant among the analyzed compounds of *M. pulegium*. Pulegone, belongs to monoterpene ketones, with a mint-like odor, which is the major component of the essential oils such as *M. pulegium*^42^. Some biological activities of pulegone including antioxidant, antimicrobial, insecticidal, anticholinesterase activity, and anti-inflammatory actions were considered by some researchers^43,44^. Franzios et al.^45^ stated that pulegone, showed strong insecticide activity among the studied constituents against *D. melanogaster*. The insecticidal activity of *M. pulegium* in the current study could be due to the highest rate of Pulegone in *M. pulegium*. Similarly, Heydarzaed et al.^31^ reported the highest amount of Pulegone in *M. pulegium*. Regardless the difference in the amounts of composition of *M. pulegium* reported in the present study, the same compounds have been reported previously^31,46,47^. The variations in environmental conditions, extraction and analysis methods of EO, plant used parts, growth stages of used plant and genetic factors could be effect on the type and amount of compounds in a plant^9,12,48,49^. However, in the current study, the percentage of the other compounds such as Menthone (%0.7) and Piperitenone (%0.7), 1-8, cineole (%8.35) were obtained in lower concentrations, their insecticidal activity have been reported by Boukhebti et al.^50^.

One promising application of *M.pulegium* essential oil is in the form of nanoformulation. In general, nanoformulations are defined as formulations with droplet sizes between 20 and 200 nanometers^51^. Characterization and determination of the nano-scald of *M. pulegium* EO is verified by using TEM, SEM, and DLS analysis. The size of *M. pulegium* oils’ nanoemulsion particle in scanning electron microscopy (SEM) was approximately less than 200 nm. Also, obtained data from TEM was coincidence with our DLS analyses. Similarly, Heydarzade et al.^31^ stated that the nano-scaled of *M. pulegium* was spherical in shape and the average size of them was around 100 and 98.99 nm using TEM and DLS instruments, respectively. Sharifiyan et al.^9^ reported that, the nanoparticle size of *M. piperita* was around 179.74nm which was higher than those of *M. pulegium* (156.40 nm) in our study. Differences in the size of nano particles and their shapes could be due to the preparation method, the type of used polymer, type of essential oil species^13^, formulation compounds (presence of surfactant), and production technique^31^.

Our results showed that lethal (LC_50_) concentration of nanoformulation essential oil had a greater effect on the adult stage of greenhouse whitefly than similar concentration (LC_50_) of the pure essential oil tested. The contact toxicity results illustrated that, the insecticidal activity increases with increasing in concentrations either *M. pulegium* oil or its nanoformulation. Also, the adults of *T. vaporariorum* were more susceptible to form of nanoformulation *M. pulegium* than pure form of essential oil after 24 h exposure. In the current study, the insecticidal activity of *M. pulegium* and its NF were more effective than those of *M. piperita* on *T. vaporariorum*^9^. It may be due to the nanoparticle size^52^. So, in our study, the obtained nanoparticle size of *M. pulegium* (156.40 nm) was smaller than those of *M. piperita* (179.74 nm)^9^. Subsequently, a smaller amount of active ingredient per area would be sufficient for application, and it can be provided sustained delivery of active ingredients which may remain effective for extended periods^52^. Also, The LC_50_ of *M. pulegium* in this research was found to be more effective on adults of *T. vaporariorum* than similar treatments on *Tribolium castaneum* Herbst (LC_50_: 7.939 µl/ml)^31^. However, Ramzi et al.^12^, reported the LC_50_ (72.94 and µl/L air,) of *M. pulegium* against *C. pipiens* after 24 h. This variation could be due to differences in the bioassay method, the species of the insect pest, developmental stages of insect, and the exposure time^12^.

Since, one of the principal aims of this work was to explore the possible lethal and sublethal effects of treatments on the life table parameters of the greenhouse whitefly over 24 h exposure, the lethal time was not consider in this research. The longer exposure time, may be increased the effectiveness of nanoformulation by increasing the solubility of the nanoformulation *M. pulegium*. However, the lethal time and stability of either pure or nanoformulation are another important subject that would be better to investigate. Finally, by reducing the dose requirement, the cost of application can be also reduced^53^. El-Medany et al.^54^ estimated the lethal concentrations (LC_50_) values of *M. pulegium* nanoemoulsion 6.645 % and 7.898 % after 48 h against *Pectinophora gossypiella* Saunders and *Earias insulana* Boisduval, respectively.

It is well known that botanic pesticides, essential oils, not only have lethal effects on insect pests in short-term, but also, they have sublethal effects in long-term by prolonging the development time, reduction in adult longevity, and fecundity on F1 generation of insect pests^9,12^. Our results demonstrated that exposure of the adults to sublethal concentration of nanoformulation was more effective than pure essential oil of *M. pulegium* on biological parameters and had noticeable effects on their offspring’s population growth rate against greenhouse whitefly. This was reflected in the combined effect exposure had on their developmental time, survival rate, first reproductive age, and fecundity, as well as the population growth parameters. In this study, the fecundity of *M. pulegium* decreased in nanoformulation treatment compared to another treatments. This finding is similar with flupyradifurone, as a novel botanical insecticide, *Aphis gossypii* (Hemiptera: Aphididae)^40^.

The population growth parameters (e. g. *R*_0_, *r* and λ) were significantly lower in the progeny of nanoformulation -treated parents than in the control group. In the current study, the obtained sublethal effects of NF *M. pulegium* on biological traits and population growth parameters of *T. vaporariorum* were more effective and lower than those of *M. piperita* NF^9^. The effectiveness of nanoformulation may be due the nano-scale structure and slow release of nanoformulation in which, the volatile properties of *M. pulegium* ingredients have been conserved during application^52^. This finding verified that *M. pulegium* nanoformulation was more effective than nanoformulated essential oil of *M. piperita*^9^ and could be a promising natural pesticide against *T. vaporariorum*.

In conclusion, our study demonstrated that *M. pulegium* essential oil and its nanoformulation have potent bioactivity against the greenhouse whitefly, *T. vaporariorum*, under laboratory conditions. This plant caused adverse effects on the biology and population growth parameters of greenhouse whitefly. The results also suggest that the nanoformulation may have enhanced efficacy compared to the pure essential oil. These findings highlight the potential of *M. pulegium* essential oil and its nanoformulation as eco-friendly alternatives for the management of *T. vaporariorum* in greenhouse production systems. However, the safety and side effects of examined essential oil and its nanoformulated form would be better to evaluate on non-target organisms.

### Materials and methods

The extraction of *M. pulegium* EO was carried out according to Sharifiyan et al.^9^ producer. Briefly, aerial parts of *M. pulegium* were collected from Piranshahr (36°41′44″N 45°08′48″E), West Azerbaijan, Iran during the summer 2021. The identification of *M. pulegium* was occurred by Dr. Larti, and deposited at the herbarium at Agricultural Research, Education and Extension Organization (AREEO), West Azerbaijan, Urmia, Iran with voucher specimen 11052. The gathered specimens were dried in shade, then the hydrodistillation (50 g/650 mL) method was done for 3 h using a Clevenger instrument (J3230, Sina glass, Tehran, Iran). Afterward, the obtained solution was incubated in darkness at room temperature for 24 h to obtain maximum essential oil extraction. Anhydrous sodium sulfate (Na_2_SO_4_) was used to dehydrate the EO. The distilled essential oil was transferred into darkness glass vials and stored at 4°C until being used in the experiments.

### Characterization of chemical constituents of *M. pulegium* by GC‑MS

Analysis of chemical constituents of *M. pulegium* were done at phytochemistry laboratory in Research Institute of Forests and Rangelands, Tehran, Iran. *M. pulegium* essential oil was analyzed by an Agilent 7890 gas chromatograph coupled with a 5975A mass spectrometry (GC-MS) using a flame ionization detector (FID) and BP-5 MS (non-polar) capillary column (30 m × 0.25 mm; film thickness 0.25 μm). The beginning oven temperature was set at 80 °C and kept for 3 min, and then increased with 8 °C/min intervals to reach up to 180°C for 10 min. Helium gas was selected as a carrier gas at a flow rate of 1 ml per min. The electron impact (EI) for ionization was 70 eV. The injector was set in a split mode, with the mass-to-charge ratio (m/z) of 40–500 m/z. The identification of components was obtained by comparing their mass spectral fragmentation patterns and linear retention indices with those described in the MS computer library^55^.

### Preparation of nanoformulation of *M. pulegium* essential oil

The synthetize of the emulsion contained nanocapsulated essential oil carried out according to our previous study^9^ using the self-assembly method and a high-speed homogenizer. The components of the aforementioned formulation were included polyethylene glycol 4000 (as a polymer), Lauryl-myristyl alcohol ethoxylates (KELAs) 3 and 7 M (as an emulsifier with hydrophilic property), Glycerol (as a cross-linker), and acetic acid (pH=3)^56,57^. For this purpose, initially to make the aqueous-based solvent of essential oil based on a new formula, 10 g of maleic anhydride was mixed with 30 g of 2-ethylhexanol and 3 ml of 98% sulfuric acid, which left for 3 h at a speed of 400 rpm to be mixed (Overhead stirres; Heidolph-TQRQUE, Germany). The resulting product was washed twice with 1 g of sodium bicarbonate to separate the two phases. The separated organic phase was left to be stirred for two hours at a speed of 500 rpm. Afterwards, the resulting material was mixed with 1 g of sodium sulfate and homogenized for 5 h at a speed of 400 rpm. The amount used in the final formulation is approximately (30-40% w/w). Next, the obtained solution was mixed with 5% soybean oil, as a synergist, 10% essential oil and a polyethylene glycol biopolymer solvent (1–5%) and stirrer at a speed of 500 rpm for 1 h. Then, the Lauryl myristyl alcohol ethoxylates (1-3% w/w) added to the mixture and homogenized at a speed of 1000 rpm for 20 min. After that, by adding Glycerol as a cross-linker (1–3% w/w) to the obtained solution, it let to stirrer at a 1000 rpm for 30 min. Finally, acetic acid (pH=3) was added to the solution drop wisely to obtain the neutral formulation (pH=6–7) and left to stirrer at 1200 rpm for 30 min to create nanoparticles of capsulated essential oil in a stable emulsion.

### Morphology, nanoparticle size and encapsulation efficiency (EE) of nanoformulation

Transmission Electron Microscopy (TEM) was carried out to visualize the shape and size of the nano-scaled emulsion droplets. First, a drop of the nano-scaled emulsion dispersion in distilled water was dripped onto a carbon- coated copper grid and dried at room temperature. Then, it was subjected to the TEM device (TEM, Zeiss-EM 10C-100KV, Oberkochen, Germany). Particle size and polydispersity index (PDI) of nanoemulsion was estimated by dynamic light scattering (DLS) (Nuremberg, Germany). Scanning electron microscopy (SEM) (TESCAN, Model: MIRA3) was used to measure the particle size of nanoformulation particles. The encapsulation efficiency was conducted according to Heydarzade et al.^31^ method by constructing the standard curve using the pulegone (% 66) as the most important constituent of *M*. *pulegium* EO.

### *Trialeurodes vaporariorum* rearing conditions

The original cohort of *T. vaporariorum* were collected using an aspirator from the greenhouse at Iranian Research Institute of Plant Protection, Tehran, Iran. In order to obtain the same aged of adults to use in experiments, 20–30 adults of *T. vaporariorum* were transferred into the ventilated plastic cages (8 cm diameter × 9 cm height) on the tomato plants (Rubi variety). After 24 h, the adults were removed, and the nymphs were monitored until they reached the desired stage for carrying out the bioassays. The colony of *T. vaporariorum* was reared in the greenhouse conditions (at 27±2°C and 60±10 % R. H. and a photoperiod of 16:8 L: D).

### Bioassays

Seven concentrations (500, 1000, 2000, 3000, 5000, 10,000 and 11,000 ppm), were selected to do preliminary bioassay tests for each treatment, in which the mortality ranges were considered between 10-90% for EO and its NF^58^. The ranges of final concentrations for the pure EO and its NF were obtained (900–10,600 ppm) and (700–8000 ppm), respectively. Due to the providing uniform treated area on the leaf surface, the dipping method was used in experiments^59^ For this purpose, the tomato leaves containing ten adults were dipped in 500 ml of each concentration of *M. pulegium* essential oil and its nanoformulation for 10 s and kept in Petri dish (8 cm in diameter). Each concentration was replicated four times. Ethanol 5% (Merck, Germany) was used as the essential oil solvent. Ethanol and nanoformulation without essential oil were selected as controls for EO and its nanoformulation, respectively. Mortality was recorded after 24 h. These experiments were carried out under greenhouse conditions as mentioned earlier. All of the used tomato leaves were in the same growth stage during experiments.

### Sublethal effects of* M. pulegium* essential oil and nanoformulation on life table parameters

The sublethal effects (LC_25_) of *M. pulegium* (1724.526 ppm) and its nanoformulation (779.439 ppm) were studied on the life history and population growth rate of *T. vaporariorum*. Briefly, tomato leaflets containing thirty adults were dipped in aforementioned sublethal concentrations (LC_25_) of treatments for 10s^53^. Then, the treated leaflets were transferred into Petri dishes (8 cm diameter) (three replicates). All adult insects were removed after 24 h, and the sixty numbers of the same aged eggs were used for each treatment and control. Afterward, the eggs were transferred individually into the ventilated Petri dishes (8 cm diameter). The leaves in each container replaced with fresh ones daily. The development and survival of preadult stages were recorded for each individual daily. After adult emergence, they were differentiated sexually by Gerling and Sinai^60^ method. Each pair was transferred in a transparent plastic container (11.5 cm in diameter ×5.5 8cm in height) containing tomato leave. The total prereproductive period (TPRP), adult prereproductive period (APRP), the number of eggs produced, number of reproductive days, adult longevity (in days), and total lifespan of the progeny of the treated parents were recorded daily.

### Statistical analysis

The LC values were determined by the Polo-Plus software (2002) using probit analysis, in which the insecticide bioassays (quantal response data) were analyzed by the techniques of probit. Also, it is able to compare the dosage-response lines by means of likelihood ratio tests. The parameters of life table were analyzed using age-stage two-sex life table^37,61^. The age-stage specific survival rate (S_*xj*_; where *x* = age, and *j* = stage), the age-stage specific fecundity (*f*_*xj*_), cumulative net reproductive rate (*R*_*x*_) values, the mean fecundity (*F*) (i.e. the contribution of an individual of age *x* and stage *j* to the future population), the age-specific survival rate (*l*_*x*_), the age-specific fecundity (*m*_*x*_), and the population parameters including the intrinsic rate of increase (*r*), the finite rate of increase (λ), the net reproductive rate (*R*_0_), and the mean generation time (*T*) were calculated according to Chi^37^ method. The bootstrap technique with 100,000 iterations was used to estimate the variance and standard errors of the biological and population parameters^62^. Also, the paired bootstrap test at a 5% significance level based on the confidence interval of differences was used to analyze the differences among treatments. Either bootstrap method or paired bootstrap test are included in the computer program TWOSEX–MSChar^63^.

## Data Availability

All data generated or analyzed during this study are included in this published article

## References

[1] Savary, S. et al. The global burden of pathogens and pests on major food crops. *Nat. Ecol. Evol.* **3**(3), 430–439 (2019). 30718852 10.1038/s41559-018-0793-y

[2] Oftadeh, M., Sendi, J. J. & Ebadollahi, A. Toxicity and deleterious effects of *Artemisia annua* essential oil extracts on mulberry pyralid (*Glyphodes pyloalis*). *Pestic. Biochem. Physiol* **170**, 104702 (2020). 32980062 10.1016/j.pestbp.2020.104702

[3] Senthil-Nathan, S. A. review of resistance mechanisms of synthetic insecticides and botanicals, phytochemicals, and essential oils as alternative larvicidal agents against mosquitoes. *Front. physiol.* **10**, 1591 (2020). 32158396 10.3389/fphys.2019.01591 PMC7052130

[4] Evans GA. Host plant list of the whiteflies (Aleyrodidae) of the world. *USDA/Animal Plant Health Inspection Service (APHIS)*. 070611 (2007).

[5] Rapisarda, C. & Longo, S. First report from Sicily (Italy) of the orange spiny whitefly, *Aleurocanthus spiniferus* (Quaintance) (Hemiptera: Aleyrodidae), and its potential risk for the Italian citrus industry. *EPPO Bull.* **51**(2), 329–332 (2021).

[6] Pym, A. et al. Host plant adaptation in the polyphagous whitefly, *Trialeurodes vaporariorum*, is associated with transcriptional plasticity and altered sensitivity to insecticides. *BMC Genom.* **20**, 1–19 (2019). 10.1186/s12864-019-6397-3 PMC6923851 31856729

[7] Gamarra, H., Sporleder, M., Carhuapoma, P., Kroschel, J. & Kreuze, J. A temperature-dependent phenology model for the greenhouse whitefly *Trialeurodes vaporariorum* (Hemiptera: Aleyrodidae). *Virus Res.* **289**, 198107 (2020). 32800806 10.1016/j.virusres.2020.198107 PMC7569604

[8] Mazzoni V, Fattoruso V, Anfora G, Pavlovcic P. Vibrational communication of the greenhouse whitefly *Trialeurodes vaporariorum* (Westwood) (Homoptera: Aleyrodidae). In *ICE 2022: XXVI International Congress of Entomology: Entomology for our planet, Helsinki, Finland, July 17-22. *511, FI. (2022).

[9] Sharifiyan, M., Mehrkhou, F. & Negahban, M. Lethal and sublethal effects of *Mentha piperita* L. and its nanoeformulation form on the biological and population growth parameters of *Trialeurodes vaporariorum* (Westwood) under laboratory conditions. *J. Entomol. Soc. Iran.* **44**(1), 25–41 (2024).

[10] Bayramzadeh, N., Mehrkhou, F., Pourmirza, A. A. & Mahmoudian, M. Fumigant toxicity of two nano-capsulated essential oils with sublethal rate of phosphine against three stored-product pests. *J. Agric. Sci. Technol.* **21**(4), 857–872 (2019).

[11] Kalyabina, V. P., Esimbekova, E. N., Kopylova, K. V. & Kratasyuk, V. A. Pesticides: formulants, distribution pathways and effects on human health–a review. *Toxicol. Rep.* **8**, 1179–1192 (2021). 34150527 10.1016/j.toxrep.2021.06.004 PMC8193068

[12] Ramzi, A. et al. Insecticidal effect of wild-grown *Mentha pulegium* and *Rosmarinus officinalis* essential oils and their main monoterpenes against *Culex pipiens* (Diptera: Culicidae). *Plants* **11**(9), 1193 (2022). 35567194 10.3390/plants11091193 PMC9105606

[13] Oftadeh, M., Sendi, J. J., Ebadollahi, A., Setzer, W. N. & Krutmuang, P. Mulberry protection through flowering-stage essential oil of *Artemisia annua* against the lesser mulberry pyralid, *Glyphodes pyloalis* Walker. *Foods* **10**(2), 210 (2021). 33498594 10.3390/foods10020210 PMC7909524

[14] Grzanka, M., Sobiech, Ł, Stuper-Szablewska, K., Danielewicz, J. & Skrzypczak, G. Effect of selected essential oils on the efficacy of volunteer oilseed rape control and phytotoxicity in maize plants. *Chil. J. Agric. Res* **82**(1), 88–96 (2022).

[15] Kačániová, M. et al. Assessment of *Ocimum basilicum* essential oil anti-insect activity and antimicrobial protection in fruit and vegetable quality. *Plants* **11**(8), 1030 (2022). 35448757 10.3390/plants11081030 PMC9031667

[16] Bartwal, A., Mall, R., Lohani, P., Guru, S. K. & Arora, S. Role of secondary metabolites and brassinosteroids in plant defense against environmental stresses. *J. Plant Growth Regul.* **32**, 216–232 (2013).

[17] Isman, M. B. Commercial development of plant essential oils and their constituents as active ingredients in bioinsecticides. *Phytochem. Rev.* **19**, 235–241 (2020).

[18] Ebadollahi, A., Jalali Sendi, J., Ziaee, M. & Krutmuang, P. Acaricidal, insecticidal, and nematicidal efficiency of essential oils isolated from the Satureja genus. *Int. J. Environ. Res. Public Health* **18**(11), 6050 (2021). 34199797 10.3390/ijerph18116050 PMC8200103

[19] Ikbal, C. & Pavela, R. Essential oils as active ingredients of botanical insecticides against aphids. *J. Pest Sci.* **92**, 971–986 (2019).

[20] Ebadollahi, A., Ziaee, M. & Palla, F. Essential oils extracted from different species of the Lamiaceae plant family as prospective bioagents against several detrimental pests. *Molecules* **25**(7), 1556 (2020). 32231104 10.3390/molecules25071556 PMC7180760

[21] Mohan, M., Haider, S. Z., Andola, H. C. & Purohit, V. K. Essential oils as green pesticides: For sustainable agriculture. *Res. J. Pharm. Biol. Chem.* **2**, 100–106 (2011).

[22] Kumar, P., Mishra, S., Malik, A. & Satya, S. Insecticidal properties of Mentha species: a review. *Ind. Crops Prod.* **34**(1), 802–817 (2011).

[23] Choupani, A., Shojaeiyan, A. & Maleki, M. Genetic relationships of Iranian endemic mint species, *Mentha mozaffariani* Jamzad and some other mint species revealed by ISSR markers. *Biotechnol. J. Biotech. Comput. Biol. Bionanotech.* **100**(1), 19–28 (2019).

[24] Turek, C. & Stintzing, F. C. Stability of essential oils: a review. *Compr. Rev. Food Sci. Food Saf.* **12**(1), 40–53 (2013).

[25] Ebadollahi, A., Sendi, J. J. & Aliakbar, A. Efficacy of nanoencapsulated *Thymus eriocalyx* and *Thymus kotschyanus* essential oils by a mesoporous material MCM-41 against *Tetranychus urticae* (Acari: Tetranychidae). *J. Econ. Entomol.* **110**(6), 2413–2420 (2017). 29029248 10.1093/jee/tox234

[26] Silva, B. D., Rosário, D. K. A., Weitz, D. A. & Conte-Junior, C. A. Essential oil nanoemulsions: Properties, development, and application in meat and meat products. *Trends Food Sci.* **121**, 1–13 (2022).

[27] Adak, T. et al. Nanoemulsion of eucalyptus oil: An alternative to synthetic pesticides against two major storage insects (*Sitophilus oryzae* (L.) and *Tribolium castaneum* (Herbst)) of rice. *Ind. Crops Prod.* **143**, 111849 (2020).

[28] Kavallieratos, N. G. et al. The type of grain counts: effectiveness of three essential oil-based nanoemulsions against *Sitophilus oryzae*. *Plants* **12**(4), 813 (2023). 36840161 10.3390/plants12040813 PMC9962515

[29] Pavlidou, V. et al. Insecticidal and genotoxic effects of essential oils of Greek sage, Salvia fruticosa, and mint, *Mentha pulegium*, on *Drosophila melanogaster* and *Bactrocera oleae* (Diptera: Tephritidae). *J. Agric. Urban Entomol.* **21**(1), 39–49 (2004).

[30] Behi, F., Bachrouch, O. & Boukhris-Bouhachem, S. Insecticidal activities of *Mentha pulegium* L., and *Pistacia lentiscus* L., essential oils against two citrus aphids *Aphis spiraecola* Patch and Aphis gossypii Glover. *J. Essent. Oil-Bear Plants* **22**(2), 516–525 (2019).

[31] Heydarzade, A., Valizadegan, O., Negahban, M. & Mehrkhou, F. Efficacy of *Mentha spicata* and *Mentha pulegium* essential oil nanoformulation on mortality and physiology of *Tribolium castaneum* (Col.: Tenebrionidae). *J. Crop Protect.* **8**(4), 501–520 (2019).

[32] Costa, T. L. et al. Lethal and sublethal effects of an essential oil-based emulsion of Patchouli, *Pogostemon cablin* (Lamiaceae), on the tomato leafminer. *Agriculture* **13**, 1540 (2023).

[33] França, S. M., Breda, M. O., Barbosa, D. R. S., Araujo, A. M. N. & Guedes, C. A. The sublethal effects of insecticides in insects. In *Biological Control Pest Vector Insect* (ed. Shields, V. D. C.) 23–39 (IntechOpen, 2017).

[34] Stark, J. D. & Banks, J. E. Population-level effects of pesticides and other toxicants on arthropods. *Annu. Rev. Entomol.* **48**(1), 505–519 (2003). 12221038 10.1146/annurev.ento.48.091801.112621

[35] Mahmoodi, L., Mehrkhou, F., Guz, N., Forouzan, M. & Atlihan, R. Sublethal effects of three insecticides on fitness parameters and population projection of *Brevicoryne brassicae* (Hemiptera: Aphididae). *J. Econ. Entomol.* **113**(6), 2713–2722 (2020). 32918545 10.1093/jee/toaa193

[36] Lashkari, M. R., Sahragard, A. & Ghadamyari, M. Sublethal effects of imidacloprid and pymetrozine on population growth parameters of cabbage aphid, *Brevicoryne brassicae* on rapeseed, *Brassica napus* L.. *Insect Sci.* **14**(3), 207–212 (2007).

[37] Chi, H. Life-table analysis incorporating both sexes and variable development rates among individuals. *Environ. Entomol.* **17**(1), 26–34 (1988).

[38] Christopher Cutler, G., Ramanaidu, K., Astatkie, T. & Isman, M. B. Green peach aphid, *Myzus persicae* (Hemiptera: Aphididae), reproduction during exposure to sublethal concentrations of imidacloprid and azadirachtin. *Pest Manag. Sci.* **65**(2), 205–209 (2009). 19089851 10.1002/ps.1669

[39] Saska, P. et al. Treatment by glyphosate-based herbicide alters life history parameters of the rose-grain aphid *Metopolophium dirhodum*. *Sci. Rep.* **6**(1), 27801 (2016). 27302015 10.1038/srep27801 PMC4908594

[40] Liang, P. Z. et al. Toxicity and sublethal effects of flupyradifurone, a novel butenolide insecticide, on the development and fecundity of *Aphis gossypii* (Hemiptera: Aphididae). *J. Econ. Entomol.* **112**(2), 852–858 (2019). 30590572 10.1093/jee/toy381

[41] Koul, O., Walia, S. & Dhaliwal, G. S. Essential oils as green pesticides: potential and constraints. *Biopestic. Int.* **4**(1), 63–84 (2008).

[42] Noma, Y. & Asakawa, Y. Biotransformation of monoterpenoids. In: Reference module in chemistry, molecular sciences and chemical engineering. *Comprehen. Nat. Products II*** 3,** 669-801 (2010).

[43] Ramadan, G. R. et al. Terpenoids, DEET and short chain fatty acids as toxicants and repellents for *Rhyzopertha dominica* (Coleoptera: Bostrichidae) and *Lasioderma serricorne* (Coleoptera: Ptinidae). *J. Stored Prod. Res.* **87**, 101610 (2020).

[44] Roy, A., Park, H. J., Abdul, Q. A., Jung, H. A. & Choi, J. S. Pulegone exhibits anti-inflammatory activities through the regulation of NF-κB and Nrf-2 Signaling pathways in LPS-stimulated RAW 264.7 cells. *Nat. Prod. Sci.* **24**(1), 28–35 (2018).

[45] Franzios, G. et al. Insecticidal and genotoxic activities of mint essential oils. *J. Agric. Food Chem.* **45**(7), 2690–2694 (1997).

[46] Dhifi, W., Jelali, N., Mnif, W., Litaiem, M. & Hamdi, N. Chemical composition of the essential oil of Mentha spicata L. from Tunisia and its biological activities. *J. Food Biochem.* **37**(3), 362–368 (2013).

[47] Brahmi, F. et al. Chemical composition and in vitro antimicrobial, insecticidal and antioxidant activities of the essential oils of Mentha pulegium L. and Mentha rotundifolia (L.) Huds growing in Algeria. *Ind. Crops Prod.* **88**, 96–105 (2016).

[48] Grdiša, M. et al. Accumulation patterns of six pyrethrin compounds across the flower developmental stages—comparative analysis in six natural *Dalmatian pyrethrum* populations. *Agronomy* **12**(2), 252 (2022).

[49] Chaudhry, S. & Sidhu, G. P. S. Climate change regulated abiotic stress mechanisms in plants: A comprehensive review. *Plant Cell Rep.* **41**(1), 1–31 (2022). 34351488 10.1007/s00299-021-02759-5

[50] Boukhebti, H. et al. Chemical composition and antibacterial activity of Mentha pulegium L. and Mentha spicata L. essential oils. *Der Pharm. Lett.* **3**, 267–275 (2011).

[51] Hadidi, M., Boostani, S. & Jafari, S. M. Pea proteins as emerging biopolymers for the emulsification and encapsulation of food bioactives. *Food Hydrocoll.* **126**, 107474 (2022).

[52] Singh, R. et al. Etched multicore fiber sensor using copper oxide and gold nanoparticles decorated graphene oxide structure for cancer cells detection. *Biosens. Bioelectron.* **168**, 112557 (2020). 32877781 10.1016/j.bios.2020.112557

[53] Shekhar, S., Sharma, S., Kumar, A., Taneja, A. & Sharma, B. The framework of nanopesticides: a paradigm in biodiversity. *Mater. Adv.* **2**(20), 6569–6588 (2021).

[54] El-Medany, W. A., El-Shennawy, R. M. & Kandil, M. A. S. Characterization of Green Mentha pulegium (L) oil nanotechnology and adverse effect on two cotton bollworms, *Pectinophora gossypiella* (Saund) and *Earias insulana* (Boisd). *Egypt. Acad. J. Biol. Sci. F. Toxicol. Pest Cont.* **14**(2), 235–248 (2022).

[55] Adams, R. P. *Identification of Essential Oil Components by Gas Chromatography/Mass Spectrometry* 5th edn. (Texensis Publishing, 2017).

[56] Constantinides PP. Microemulsions containing pharmaceutical compositions. EP0684832A1. (1993).

[57] Thonggoom, O., Punrattanasin, N., Srisawang, N., Promawan, N. & Thonggoom, R. In vitro controlled release of clove essential oil in self-assembly of amphiphilic polyethylene glycol-block-polycaprolactone. *J. Microcapsul.* **33**(3), 239–248 (2016). 10.3109/02652048.2016.1156173 26988617

[58] Robertson, E. L. & Liber, K. Bioassays with caged *Hyalella azteca* to determine in situ toxicity downstream of two Saskatchewan, Canada, uranium operations. *Environ. Toxicol. Chem.* **26**(11), 2345–2355 (2007). 17941726 10.1897/06-489R.1

[59] Sohrabi, F., Shishehbor, P., Saber, M. & Mosaddegh, M. S. Lethal and sublethal effects of buprofezin and imidacloprid on *Bemisia tabaci* (Hemiptera: Aleyrodidae). *Crop Protect.* **30**(9), 1190–1195 (2011).

[60] Gerling, D. & Sinai, P. Buprofezin effects on two parasitoid species of whitefly (Homoptera: Aleyrodidae). *J. Econ. Entomol.* **87**(4), 842–846 (1994).

[61] Chi, H. S. I. N. & Liu, H. Two new methods for the study of insect population ecology. *Bull. Inst. Zool. Acad. Sin.* **24**(2), 225–240 (1985).

[62] Effron B, Tibshirani RJ. An introduction to the bootstrap. Chapman & Hall/CRC, Monographs on Statistics and Applied Probability, New York. (1993).

[63] Chi H. TWOSEX-MSChart: a computer program for the age-stage, two-sex life table analysis. Accessed on 25 May 2005. (2005).

